# A Study of Saliva and Its Action on Tooth Enamel in Reference to Its Hardening and Softening*Read in the Section on Stomatology of the American Medical Association, at the Sixty-Third Annual Session, held at Atlantic City, June, 1912.

**Published:** 1913-03-15

**Authors:** Joseph Head

**Affiliations:** Philadelphia


### A STUDY OF SALIVA AND ITS ACTION ON TOOTH ENAMEL IN REFERENCE TO ITS HARDENING AND SOFTENING.*

JOSEPH HEAD, M.D., D.D.S., PHILADELPHIA.

Enamel softening has been considered to be necessarily associated with roughening of the surface, loss of luster and dissolution of the cemental substance that binds the enamel rods together; and whenever enamel decalcification is associated with decay of dentine this conception of enamel softening is only too accurate. But this conception seems to be only a part of the story, so to speak—the final part rather than the complete process of enamel degeneration.

For instance, 1 to 1,000 lactic acid and water at mouth temperature will cut tooth enamel in thirty minutes with a rough, white surface. A tooth placed in 1 to 500 lactic acid and some salivas will be unharmed. A tooth placed in this solution made with other salivas, after three or four days, may show the enamel perfectly smooth and to all appearances normal, and yet it can readily be pared to a slight distance with a lancet. A 1 to 500 saliva and lactic acid solution has an extremely acid taste and instantly turns litmus brilliantly red. A 1 to 20,000 lactic acid and water solution will, at the end of three or four days, leave the tooth enamel unharmed to all appearances, and yet the outer surface of the enamel will, with the edge of the lancet, be found to have distinctly softened. A 1 to 500 lactic acid solution with my saliva, although the solution has a decidedly acid taste, will produce this identical effect. The appearance of the enamel is unchanged but distinctly softened to the cut of a lancet, although before the tooth has been immersed the lancet is unable to make any impression on it.

*Read in the Section on Stomatology of the American Medical Association, at the Sixty-Third Annual Session, held at Atlantic City, June, 1912.

The action of water solutions of acid calcium phosphate and acid sodium phosphate on the teeth has been mentioned by Kirk, stress being laid on the fact that these salts cause smooth white decalcification. As a matter of fact, many acids in water solutions cause smooth primary softening, if the solution is sufficiently weak. Saliva ordinarily restrains the action of most acids up to a certain point, and then begins with the smooth decalcification and ends with the same rough white decalcification that we find in a water solution. The saliva solution is ordinarily from ten to twenty times weaker in its action on enamel than is a water solution of the same acid strength. The acid sodium phosphate and acid calcium phosphate are, however, intensely interesting in their action, not only in water solutions but in saliva solutions. Acid calcium phosphate 1 to 5,000 water solution, and acid sodium phospate 1 to 20,000 water solution at the end of two days, will turn tooth enamel into a cloudy, pearly white, with a clear smooth surface. This surface is distinctly softened to the cut of a lancet. If, however, these teeth are removed from the solutions before the process advances too far, the cloudy appearance will in time disappear, and the tooth enamel will resume its normal appearance. This phenomenon occurred many times, but once when I retested the hardness of these teeth with a lancet I found that not only had the white color disappeared but the superficial softening of the enamel had disappeared also. At first I attributed this disappearance of the softening of the enamel to previous faulty observations, but the same phenomenon occurred in my lactic acid test. A tooth that had been placed in a 1 to 20,000 lactic acid watery solution, at the end of a few days was slightly softened to the cut of the lancet, but a month or so later the lancet would not cut it. It was then that the possibility was made apparent that partially softened smooth enamel could reharden itself if the decalcification had not progressed too far.

I then made the following test: Two sound teeth with enamel impervious to the lancet were each placed in a lobe of a naval orange. These lobes were each placed in a bottle with a few drops of ether to prevent fermentation, and kept at body temperature for two days. At the end of that time the teeth were removed and examined. The lobe around one of the teeth had fermented, the lobe around the other had not. The tooth in the fermented lobe showed a smooth, white, translucent area of decalcification, running from the cutting edge to about one-third of the distance to the neck. The rest of the enamel was normal to all appearances, and yet the surface, both of the white and the apparently normal enamel, was readily pared with a lancet. The lobe in which the other tooth was embedded had not fermented, and the enamel also seemed normal, but here again the outer surface of the tooth enamel was distinctly softened. These teeth were then washed in water and kept in some of my saliva at body temperature for two weeks. At the end of five days there was a decided rehardening of the enamel surface, and the white area of decalcification on the tooth mentioned had half disappeared. At the end of ten days the enamel could no longer be scratched with a lancet; at the end of two weeks the white spot of decalcification had almost if not entirely disappeared, and both teeth appeared perfectly normal.

That saliva does posses the power of restraining enamel from carbon dioxid decalcification was proved as follows: A sound extracted tooth was placed in a sparklet or automatic soda water former, where the liquid within could be charged from a carbon dioxid cartridge. Thirty cubic centimeters of saliva were added which had been obtained by chewing rubber. The saliva was then charged with carbon dioxid and the sparklet placed in a culture oven at a temperature of 98 F. for thirty days. At the end of that time it was taken out and appeared unharmed. The tooth was then washed with a little ether and replaced in the syphon with distilled water. This water was then charged with carbon dioxid and the flask replaced in the culture oven for twenty-four hours. At the end of that time the enamel showed a chalky decalcification that could be scaled off with the finger nail.

This protective power of saliva is also exerted against lemon, orange, grape, grape fruit, strawberry, rhubarb and cherry, the special action of which was the subject of a paper[1] I wrote in 1908.

These experiments with extracted teeth, weak acid solutions and tests with a sharp lancet in the hand, while suggestive, seemed to me not sufficient to establish so reactionary a doctrine; so while morally convinced of the truth of my assertion that softened enamel could reharden, I immediately started to perfect a machine that would show in the minutest degree just how far a given force would drive a standard punch into sound enamel, partly decalcified enamel and rehardened enamel, if it did reharden, from the effects of slight decalcification. I will not dwell on the discouraging task of making good mechanics work with a scientific conscience. They would not do as they were told, and much of the adjustment had to be done by the deviser of the instrument, which from at first having an error of 1/500 of an inch, finally developed into a micro-dynamometer that could deliver 475 pounds pressure on a punch, the penetration of which could be measured up to 1/600,000 of an inch. Something approaching this accuracy was necessary as it was found experimentally that the scope of the average test lay usually within 1/10,000 of an inch. It was, however, decided to set the register so that it would measure in units of 1/300,000 of an inch, which as can readily be seen could be reduced by a table to microns or tenths of microns, one micron being equal to 1/25,000 of an inch, twelve of the machine units, therefore, being equal to one micron. The pressure was given by a mercury gauge to which the punch was attached. A micrometer screw supporting the anvil on which the specimen for testing was to be placed was strongly connected by castings and heavy

1. Read before the Harvard Odontological Society, March 7, 1908.

drawn steel rods beneath the mercury gauge and punch. The pressure was applied to the specimen by placing it on the anvil and raising the anvil by the screw up against the punch until the mercury marked the pressure desired. The penetration of the punch into the specimen was made known by a microscope equipped with a specially constructed filar micrometer, which microscope was solidly screwed to the anvil. This microscope was then adjusted and set so that it could accurately divide into the desired number of parts a glass scale attached to the punch. By this device, all error in the use of the machine was largely thrown out, since only the relation between the punch and the anvil was within the scope of measurement.

In making the various tests three punches are used: a heavy steel punch with a flat hardened circular point 1/50 of an inch in diameter; an iridium pointed punch 1/50 of an inch in diameter for testing acid erosion; it will stand up to 25 lbs. without showing compression; a diamond-pointed punch with a circular, flat point 1/50 of an inch in diameter that can be used in acid solutions and will also readily stand 100 lbs. pressure. Twenty-five pounds pressure with the diamond point will give a penetration equal to 75 lbs. with a steel point. This is no doubt due to the greater sharpness of the diamond edge over the steel.

If, therefore, the specimen is so adjusted and set under pressure that there will be no give between it and the anvil it is possible to determine accurately within, say, one-tenth of a micron, just how far the punch would penetrate under a given pressure. For instance, the specimen is carefully ground with a Reichert section grinder, so that there is a flat, large base. A small spot on the top of the enamel is ground parallel to the base. This forms the area to be tested. Any pressure exerted on the enamel spot by the punch is directly expended on the enamel, without any side give between the base of the specimen and the anvil. The specimen with parallel sides is placed on an agate slab and the slab placed on the anvil. The punch is adjusted near the edge of the ground enamel and given a pressure of 5 pounds. A reading by the microscope attached to the anvil is then taken on the scale attached to the punch. The pressure is then raised to the desired amount, say, 75 pounds, for a given time, and then the pressure is reduced again to 5 pounds and another reading taken, the diff rence on the scale representing the penetration of the punch. To shift the specimen under the point for the different measurements, the agate slab is moved, not the specimen on the slab. This eliminates variations in the spring of the specimen, since the compression of the parallel slab on the anvil under a given pressure, will always be the same.

Five or more measurements are usually made for each reading and the average taken. By this means the density and strength of normal enamel can be shown, as can also the variations from the normal enamel caused by strong acids, water solutions of acids, saliva, fresh and stale acid saliva solutions, and the recovery of enamel from the effects of a slight acid decalcification and the rehardening of its surfaces after it has been ground with a stone.

All analytical analyses of saliva heretofore have been hampered by the fact that the investigator of it first started by chemically breaking it up, thus largely destroying it as saliva. All analytical investigation of saliva from a dental point of view up to the present time, has been of little value owing to the fact that normal saliva may be alkaline to lacmoid or congo red, neutral to litmus, and acid to turmeric paper, all at the same time. How saliva reacts to lacmoid, litmus or turmeric may be purely of academic interest, but how it may react on enamel and dentine is a vital question that interests many ever that lives, and this machine has, it is hoped, made it possible for enamel to be its own indicator of how a saliva may affect it. Since the first days of physiologic study, saliva has been a most tempting field for investigation because it is the body secretion most easily obtained in a living state. It ought to be at least of equal value to the diagnostician as urine, but up to the present time it has not been. This may lie in the fact that saliva is a living substance, and urine is an effete product that lends itself to a chemical analysis that would absolutely destroy saliva as saliva. Chemical analysis that breaks up the individuality of living saliva is of little more value in showing the vital action of saliva than fried chicken would be in showing the beauties of a cock fight.

I am far from discouraging saliva analysis of any kind. Epinephrin may illustrate the living secretion of the suprarenal capsule or it may not. Salivary extracts or chemical products are interesting but inconclusively related to the living action of the secretion and, therefore, while enzymes of a curious and even therapeutic nature may be extracted from saliva, no one can prove that they justly represent the action of the living secretion unless some means are devised to make control tests with the living saliva that show the corresponding action. This machine, it is hoped, will make it possible to test on tooth enamel the acid restraining power of the various living salivas in relation to a standard water solution of acid. Salivas vary in this power. The same saliva may vary at different times, apparently according to the condition of the patient.

The conditions then are as follows: During sickness the teeth, from clinical experience, are known to decay. This may be due partly to acid medicine or lack of care, but with every allowance for these sources of error. it seems to be well established that severe or chronic illness in some way may render the teeth sensitive to deterioration. Formerly it was supposed that some product was extracted from the enamel through the blood circulation, but this is now fully disproved by the histologic knowledge that human tooth enamel is, to all intents and purposes, absolutely shut off from the blood current. If a relation between approaching sickness and a loss of acid protecting power in the saliva could be established, a beginning in the diagnostic testing of the living saliva would have been fairly inaugurated. Take for example, the test that was made on a specimen of tooth enamel that I use for an illustration. One side was ground to a flat, broad base, on the other side the enamel was ground in a small corresponding parallell plane. This was placed on the anvil of the machine and raised up against an iridium point 1/50 of an inch in diameter until a pressure of 5 pounds was reached. A reading was then taken on the micrometer scale and noted. The anvil was raised up and down repeatedly until all give between the specimen and anvil disappeared, and the 5 pounds pressure always gave the same reading. Then fresh lemon juice and water 1 to 100 was placed on the specimen around the point and the point relaxed so that the fluid could readily get underneath. At certain intervals of time the pressure of 5 pounds was tried and a reading taken which showed a loss of tooth structure as follows:

| | | | |
|---|---|---|---|
| 2 | minutes | 0.8 | micron |
| 5 | " | 1.2 | " |
| 10 | " | 1.4 | " |
| 35 | " | 2.3 | " |
| 60 | " | 2.8 | " |

Thus, in one hour there was a loss of 2.8 microns of tooth enamel. This same test was then made on the same tooth enamel with a fesh saliva solution of lemon juice, 1 to 100. This solution turned litmus red and was distinctly sour, and yet after an hour's application the enamel showed a loss of only 0.3 of a micron. We might, therefore, express the restraining factor of that saliva as 28 : 3 or 9 Numerous other tests have been made of a similar nature and all seem to give consistent figures. They almost invariably seem to prove the power of saliva to restrain the action of lemon juice on enamel. They also show that some enamels are much more resistant to decalcifying action than others.

As an illustration, another test might prove interesting: This specimen was ground longitudinally for a base along its axis and a small parallel plane ground on the enamel of the opposite side. A solution of 1 to 100 lemon juice and water applied for an hour dissolved 1.0 micron. Saliva and lemon juice, 1 to 100, applied for sixteen hours showed no loss of tooth tissue at all. It was then tested with the steel punch 1/50 of an inch for density and was found so softened that from 60 pounds to 70 pounds pressure could not be obtained. The softened enamel simply refused to support the punch under such a pressure. The specimen was then placed in fresh saliva and set in the culture oven at blood heat for seven hours, the saliva being changed every half hour. At the end of that time the tooth was again tested with the steel punch and was found to have so hardened that 75 pounds pressure was readily withstood and a various penetration noted of 3.5, 2.8, 3.2, 4.5 microns. No doubt it would have hardened up more but the various testings had so strained the specimen that it was considered advisable to continue with others. The great softening and hardening apparent in this last test is no doubt due to the enamel being very thin so that the variations were partly due to the dentine underneath. But tests on two bicuspids extracted from the same mouth for the purpose of regulation, with perfect enamel, to all appearances show conclusively that enamel has an extraordinary power of hardening and softening under various conditions, although no acid solvent at all was used.

These specimens were soaked for about ten days in a 1 to 10 dilution of liquor formaldehyde in water. They were then cut flat at the neck and 1/80 of an inch ground off the cusps of Specimen 1, and 1/50 of an inch ground off the cusps of Specimen 2. The diamond point was used at 25 pounds pressure.

Specimen 1 (buccal cusp) when freshly ground gave an average softness of 1.5 microns, the readings being: 1.1, 2.0, 1.2, 1.8, 1.8, 1.2.

Specimen 2 (buccal cusp) gave an average softness of 1.6 microns, the readings being: 1.2, 1.6, 1.6, 1.5, 2.1.

Specimen 1 (lingual cusp) gave an average penetration of 1.2 microns, the readings being: 1.3, 1.2, 1.3, 1.2, 1.0.

Specimen 2 (lingual cusp) gave an average penetration of 1.1, the readings being: 1.3, 0.9, 1.1, 1.3, 0.9.

Here in two specimens apparently of similar teeth from the same mouth, we find the buccal cusps having the same hardness with 0.1 of a micron, and the same with the lingual cusps; while the lingual and buccal cusps on the same teeth show a variation in penetration of from 0.5 to 0.3 micron respectively.

I speak of this comparison to show the consistency of the machine measurements. I shall now proceed with the tests on Specimen 1:

After being tested the specimen was placed in distilled water for three hours and again tested. The buccal cusp had softened from 0.3 micron to 1.8 microns, the measurements being: 1.4, 2.4, 3.9, 1.9. The lingual cusp had not changed showing a penetration of 1.2, microns, measurements being: 1.5, 1.3, 1.3, 1.3, 0.8.

The tooth was then placed in J. H. saliva over night

In the morning the buccal cusp showed a slight hardening of one point, to the average hardness of 1.7, the measurements being: 1.8, 1.3, 1.8, 2.2, 1.6.

The lingual cusp had hardened 0.2 to the average of 1.0, measurements being: 1.1, 1.2, 1.0, 0.8, 1.1.

The tooth was allowed to stay in J. H. saliva, at room temperature, all day (nine hours) and tested again, when the buccal cusp had hardened seven points, to the average of 1.0, measurements being: 0.9, 0.8, 1.2, 1.0, 0.9, while the lingual cusp had hardened 0.1, to the average density of 0.9, measurements being: 0.4, 0.7, 1.1, 1.3, 1.1, 0.7. In this instance, the buccal cusp started at 1.5, softened to 1.8, and hardened to 1.0. The lingual cusp started at 1.2, and stayed the same, or consistently hardened to 0.9.

It was decided to try the saliva of my assistant on this specimen.

The specimen was placed in incubator, 98 F., with A. J. F. saliva and a little ether for 36 hours, and buccal cusp showed a softening of 0.5 or an average of 1.5, measurements being: 1.7, 1.4, 1.2, 1.4, 1.5, 1.5. Lingual cusp remained the same or 0.9, measurements being: 1.0, 0.8, 0.8, 1.2, 0.6. The specimen was kept in A. J. F. saliva for a day longer, when the buccal cusp showed a further softening of 0.7, penetration being, 2.2, the measurements being: 1.4, 3.4, 1.5, 2.0, 2.6. The lingual cusp showed a softening of 0.3, having an average penetration of 1.3, measurements being: 1.6, 1.6, 1.4, 1.1, 1.0.

The specimen was then placed in a 2 per cent. dilution of liquor formaldehyde in water for sixteen hours, room temperature, when the buccal cusp was found to have hardened 1.2 to 1.0, measurements being: 1.3, 0.8, 1.0, 1.0, 0.8, and the lingual cusp had hardened from 0.1 to 1.2, measurements being: 1.4, 0.8, 1.0, 1.8, 0.9.

Specimen 2: The buccal cusp showed a hardness of 1.6, the lingual cusp a hardness of 1.3. It was placed in A. J. F. saliva and 2 per cent. ether in incubator at 90 F. for three and one-quarter hours and tested again. The buccal cusp had hardened 0.5 points to 1.1, measurements being: 0.9, 1.0, 1.2, 1.2, 1.2. The lingual cusp showed a hardening of 0.2 points, having an average of 0.9, measurements being: 0.8, 1.0, 1.3, 0.9, 0.8, 0.9. The specimen was then replaced in the same saliva in the incubator for two days and the buccal cusp showed a further hardening of 0.1

point, giving a penetration of 1.0, measurements being: 0.5, 0.8, 1.2, 1.2, 1.0, 1.2, 0.8.

The tooth was then placed in J. H. saliva, at room temperature for eight hours, when the lingual cusp showed a softening of 0.6 point or an average of 1.5, measurements being: 1.2, 1.4, 1.1, 1.9, 1.9, 1.7, 1.0.

The tooth was then placed in A. J. F. saliva, in incubator for fifteen hours, when the buccal cusp was found to have softened 3 points to 1.3. measurements being: 1.4, 1.3, 1.3, 1.2, 1.2, while the lingual cusp had hardened 5 points to 1.0, measurements being: 0.9, 0.9, 1.0, 1.1, 1.1.

The specimen was put in A. J. F. saliva in incubator and the saliva was changed every half hour. In four hours, the buccal cusp had hardened 2 points to 1.1, measurements being: 1.2, 1.2, 1.1, 1.2, 1.0, 1.2, 1.0, while the lingual cusp remained about the same, showing also a penetration of 1.1.

The specimen was then placed in A. J. F. saliva and 2 per cent. ether in incubator for twenty-four hours, when the buccal cusp was found to have softened from 0.5 points to 1.6, measurements being: 0.9, 0.8, 2.1, 1.4, 2.8, 1.6, 1.4, and the buccal cusp had softened 0.2 point to 1.3, measurements being: 1.4, 1.3, 1.2, 1.2, 1.3.

Then 5—1000 of an inch was ground off the tooth surface and the cusps were again measured to see how they would compare with the original tests. The buccal cusp showed an average penetration of 1.7, one more than the original test, measurements being: 1.7, 1.8, 1.6, 1.8, 1.6, while the lingual cusp showed exactly the same hardness, having a penetration of 1.1. The tooth was then placed in the incubator with A. J. F. saliva changed every half hour for six hours. The buccal cusp hardened 0.4 point to 1.3, measurements being: 1.9, 0.9, 1.2, 1.3, 1.0, 1.5, 1.2. The lingual cusp had softened 0.1 point to 1.2, which was practically remaining the same, measurements being: 1.2, 1.4, 1.2, 0.8, 1.2, 1.4.

The tooth was then placed in A. J. F. saliva and ether in incubator over night, when the buccal and lingual cusps showed an equal penetration of 1.3, the buccal measurements being: 1.4, 1.0, 1.5, 1.4, 1.3, and the lingual measurements being: 1.2, 1.2, 1.4, 1.2, 1.4.

This softening and hardening of enamel has a very practical bearing in relation to mouth cleansing and the use of brush and dentifrices. Enamel that in its hardest state would only show insignificant wear to dentifrice grit and friction in a temporarily softened state may, through a good brushing with grit, lose a very material amount of tooth structure. This softening and hardening may readily account for the reason why some patients, especially those fond of fruit, wear their teeth down to the gum, at a comparatively early age. The mere friction of mastication is sufficient to take off a layer of enamel softened by fruit or vinegar, which if left to itself might reharden.

I have made hundreds of tests with this machine, but as these are largely of interest to dentists, I will reserve them for another paper, where there will be ample space for the tables that should, of necessity, accompany my findings concerning the variations in the action of enamel, which variations are so extraordinary as to be almost beyond belief.

But that enamel will harden and soften within certain limits, I am convinced. And that this hardening and softening is influenced by the saliva and foodstuffs is to me beyond the question of a reasonable doubt. This action I also believe may occur in dentine, but my tests concerning this latter are not sufficiently numerous to serve as a basis for a positive assertion.—*Journal A. M. A.*

